# Computer-Automated Static, Dynamic and Cellular Bone Histomorphometry

**DOI:** 10.4172/2157-7552.S1-004

**Published:** 2012-12-24

**Authors:** Seung-Hyun Hong, Xi Jiang, Li Chen, Pujan Josh, Dong-Guk Shin, David Rowe

**Affiliations:** 1 Department of Computer Science and Engineering, University of Connecticut, Storrs, Connecticut, USA; 2 Department of Reconstructive Sciences, Biomaterials and Skeletal Development School of Dental Medicine, University of Connecticut Health Center, Farmington, Connecticut

**Keywords:** Histomorphometry, Enzymatic stains, Cryohistology, Image processing, Image analysis

## Abstract

Dynamic and cellular histomorphometry of trabeculae is the most biologically relevant way of assessing steady state bone health. Traditional measurement involves manual visual feature identification by a trained and qualified professional. Inherent with this methodology is the time and cost expenditure, as well as the subjectivity that naturally arises under human visual inspection. In this work, we propose a rapidly deployable, automated, and objective method for dynamic histomorphometry. We demonstrate that our method is highly effective in assessing cellular activities in distal femur and vertebra of mice which are injected with calcein and alizarin complexone 7 and 2 days prior to sacrifice. The mineralized bone tissues of mice are cryosectioned using a tape transfer protocol. A sequential workflow is implemented in which endogenous fluorescent signals (bone mineral, green and red mineralization lines), tartrate resistant acid phosphatase identified by ELF-97 and alkaline phosphatase identified by Fast Red are captured as individual tiled images of the section for each fluorescent color. All the images are then submitted to an image analysis pipeline that automates identification of the mineralized regions of bone and selection of a region of interest. The TRAP and AP stained images are aligned to the mineralized image using strategically placed fluorescent registration beads. Fluorescent signals are identified and are related to the trabecular surface within the ROI. Subsequently, the pipelined method computes static measurements, dynamic measurements, and cellular activities of osteoclast and osteoblast related to the trabecular surface. Our method has been applied to the distal femurs and vertebrae of 8 and 16 week old male and female C57Bl/6J mice. The histomorphometric results reveal a significantly greater bone turnover rate in female in contrast to male irrespective of age, validating similar outcomes reported by other studies.

## Introduction

Despite its importance in understanding bone pathophysiology, the traditional bone histomorphometry have some drawbacks: high cost, slow processing speed and observer-subjectivity in data quantification. In traditional bone histomorphometry, a commercial histomorphometry platform uses a computer, an input device (such as mouse or stylus pen), and a microscope. A histological sample (either frozen section or paraffin-embedded section, but mostly latter) is placed on the microscope stage attached to the computer. A technician searches for region of interest (ROI) and traces signals (bone surfaces, mineralized labels, cells, etc.) within the sample using input devices in conjunction with the computer monitor. Before tracing is performed, the input regions to query are primed by clicking appropriate tick marks. When the tracing is done, the computer calculates the parameters. The tedium of this computer-aided manual measurement in a dark room (due to using microscope) usually confines technicians to a handful of analyses per day. More importantly, there is a degree of subjectivity that technicians introduce during the quantification process. This subjectivity may be consistent within an experiment or observer, but will be prone to control error when multiple laboratories are working in conjunction. We propose a new platform as an alternative that provides an automated, cost effective (including technician time), rapid and observer-independent method for dynamic histomorphometry that overcomes the limitations of traditional methods, while promising a greater consistency and higher throughput in assessing cellular activities. One key aspect of our method has been incorporating high-throughput bone imaging, measurement and analysis into a seamless workflow to achieve speed and objectivity in data quantification. Our method has a great potential to be widely adopted by the skeletal biology research communities of tissue engineering, in-vivo repair model development, and gene knockout studies, who generally use bone histology as a research tool.

## Methods

### Ethics statement

The mouse study was done in accordance with all applicable federal, state, and institutional laws, policies, and guidelines of animal experiments and was reviewed and approved by the Animal Care Committee at University of Connecticut Health Center (protocol #2010-610).

### Cryosectioning and bone imaging

The first part in our analysis workflow is the image acquisition. The issues here are producing high quality images and assembling images in a manner that is amenable to automated analysis and comparison among known dynamic properties of the distal femur and vertebra. Here we present the six steps of performing cryosectioning and high speed bone imaging to analyze mice that are labeled with two fluorochromes and whose bone lining of osteoblasts and osteoclasts are to be assessed by quantifying the amounts of fluorescent based enzymatic stains.

#### Labeling mice and harvesting tissue

Weaned male and female C57Bl/6J mice were purchased from Jackson Labs and aged to 8 weeks and 16 weeks of age. Each group of 8 mice was administered by intraperitoneal injection Alizarin Complexone (AC) (30 mg/kg (Sigma A-3882)) at 7 days and calcein (10 mg/kg calcein (Sigma C-0875)) at 2 day prior to sacrifice by CO_2_ asphyxiation. The vertebrae and femurs are removed and the non-adherent muscle and connective tissues are removed from the bone without scraping the periosteal surfaces. The samples are placed in 10% neutralized formaldehyde (Sigma, HT501320.9) at 4° for 2–3 days with slow agitation.

#### Embedding and sectioning

One femur and 4^th^ lumbar vertebra per mouse are soaked in 30% PBS sucrose overnight and embedded in Neg-50 frozen section medium (Richard-Allan Scientific, #6502) over methylbutane chilled in dry ice. In the following day, the blocks are trimmed and sectioned using the Leica 3400 cryostat, disposable steel blade (Fisher Scientific, # 3051835) and adhesive tape transfer step (Section Lab, Co., Ltd, Toyota-gun, Hiroshima, Japan 7250301). The middle part of the bone is sliced at 5 µm thickness. It takes about 15 minutes to produce two slides with 2–4 sections per slide (sample side down) for archival. Multiple sections are taken throughout the block and checked by a light microscope to position the section of the femur to include the central vein and a mid-region of the vertebral body. These sections and the remaining tissue block and other femur are maintained at −80°C for long-term storage.

For the purpose of imaging, one of the sections is placed on a 1”×3” glass slide with the tape side down using a 2% chitosan (Sigma #C3646) solution in 0.25% acetic acid and allowed to air dry for 48 hours at 4°C frost free refrigerator. In addition, 1 µl of water containing a suspension of 6µm calibrated fluorescent bead (Molecular Probes #I-14785m green; #I-14787 red) is placed on or near the section and air-dried.

#### Exposure time

**Figure 1:**
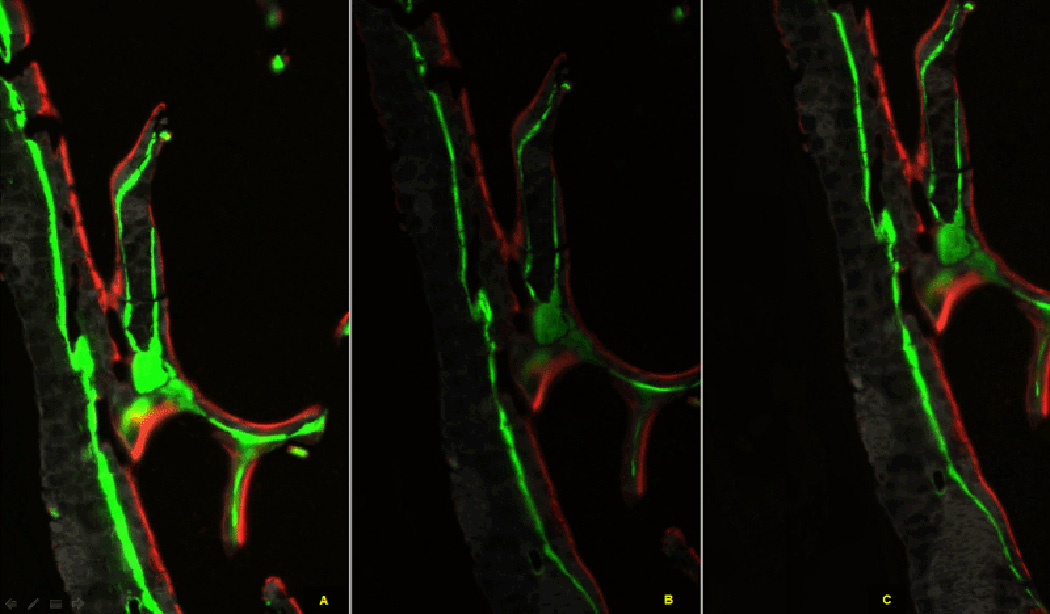
Images scanned with various exposure times. They show different signal strength and width. (A) Image with manual high exposure time. (B) Image with automatic exposure time. (C) Image with manual low exposure time.

In using microscope scanner, we find that controlling the exposure time of a camera can greatly affect the histomorphometic outcome. Figure 1 illustrates the same images scanned with three different exposure times. Figure 1A–C show the images with manual high exposure time (YFP=60 ms, TRITC=250 ms), automatic exposure time (YFP=16 ms, mCherry=96 ms), manual reduced exposure time (YFP=30 ms, TRITC=200 ms), respectively. In the high exposure, the YFP signals are broader than those of the other exposure time cases. The YFP signal width is noticeably narrow in the auto exposure. The problem is that as shown in table 1, exposure time does affect measurements. This table summarizes comparison among three exposure cases for bone surface ratio associated with YFP (G/ BS), single labeled surface ratio (sLS/LS), and labeled surface ratio (LS/ BS). In the experiments reported in this paper, we have used manually selected fixed exposure. In future studies, we plan to explore finding the optimum exposure time by experiment with alternative exposure choices and their impact on the overall study outcome.

#### Shade correction

Inhomogeneous lighting or misalignment of camera may cause a shade in the scanned image, and the fractional images or the assembled image may have multiple intensity groups in which intensity changes gradually from one side to the other side. This affects the segmentation process. In order to remedy the shade effect, a shade correction will have to be carried out. In our experiment, we have used a median filtering. The corrected image can be obtained by subtracting the median filtered image from the original image. The size of the median filter should be bigger than the size of the trabecular tissues; otherwise some bone tissues are erased by the subtraction procedure. This process also removes the minor background noise introduced from shading.

#### Imaging the mineralized section

The slide to be imaged is placed for 10 minutes in PBS, stained in a 30 mg/ml calcein blue solution (Sigma, #M1255-1G) for 30 minutes and cover slipped with 50% glycerin. The microscope used for scanning the bone section is Carl Zeiss’ Mirax Midi high speed automated image acquisition system equipped with a 20X/0.8 plan-Apochromat objective (Cat#440640-9903-000) and high resolution camera (AxioCam HRm). After the ROI is canned and the depth of Z axis for automatic focusing are set, the microscope is instructed to take a series of three images per field (DAPI filter, Chroma, #49000ET; Tetramethyl Rhodamine Iso-Thiocyanate (TRITC) filter, Chroma #49005ET; Yellow Fluorescent Protein (YFP) filter, Chroma #49003ET) to capture the mineral, calcein and AC signals, respectively.

#### Imaging osteoclasts

**Figure 2:**
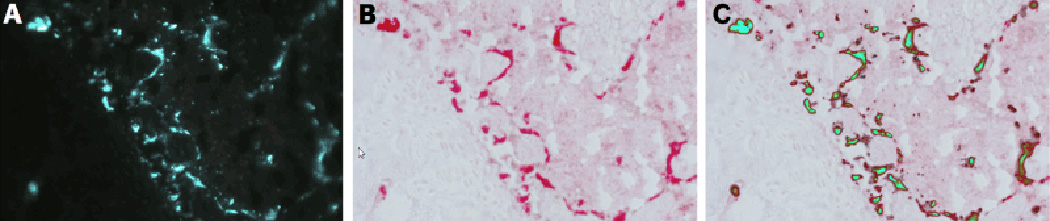
Co-localization of the fluorescent ELF-97 signal with the traditional chromogenic signal used in the TRAP enzymatic assay. (A) ELF-97 substrate; (B) Chromogenic stain based on a commercially supplied kit (Sigma387A) (C) Overlay of the two images.

As currently practiced, osteoblasts and osteoclasts are identified visually by the individual performance of the histomorphometric analysis. Because our goal is to develop an observer-independent system, we need to have a distinct fluorescent surrogate for these cells. The Tartrate Resistant Acid Phosphatase (TRAP) stain is done under acidic conditions using the fluorescent substrate (Invitrogen ELF-97 (Enzyme-Labeled Fluorescence-97) E6589) [1]. The acid rapidly removes the fluorescent mineralized labeling lines and most of the mineral within the bone section. The incubation and reagent concentrations are modified from the traditional colorimetric TRAP stains to adjust for the stronger enzyme activity of the frozen section. The TRAP signal is captured with a filter optimized for tetracycline (Chroma Technology Custom HQ409sp, 425dcxr, HQ555/30, set lot C-104285). ELF-97 was employed under acidic condition to detect TRAP activity associated with osteoclasts. Figure 2 shows good concordance of this fluorescent substrate with the colorimetric substrate utilized in traditional TRAP staining. In practice the substrate identified both mononuclear and multi-nuclear cells with TRAP activity. When the TRAP signal is overlaid with the mineralization image, a distinction between ELF-97 positive cells on the bone surface lacking a mineralization line can be made from those which are either not in association with the bone surface or those adjacent to a mineralization line. We interpret ELF-97 positive cells on the bone surface lacking a mineralization line as the fluorescent surrogate for an eroded surface.

#### Staining for osteoblasts

**Figure 3:**
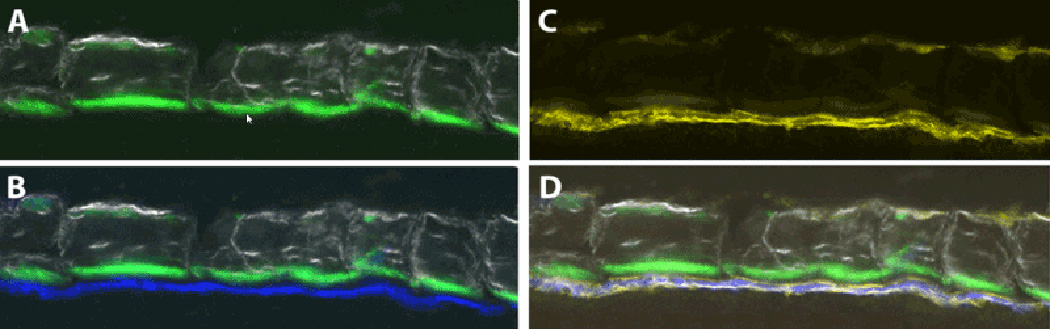
Co-localization of AP activity with areas of active and inactive bone matrix formation. Images are taken from cortical bone of a Col3.6GFPcyan reporter mouse. (A) Bone mineral with a calcein green mineralization line on the endocortical surface. (B) Same section now with the Col3.6blue signal derived from the active osteoblasts expressing the reporter. (C) Same section stained for ELF-97 and imaged in the fluorescent channel specific for its fluorescent spectrum. (D) Co-localization of all the fluorescent signals showing that the AP activity of the ELF-97 overlies the osteoblastic cells on the endosteal surface, while the weaker ELF-97 signal on the periosteal side does not have a calcein or Col3.6 reporter signal.

Alkaline Phosphatase (AP) activity is detected by the fluorescent substrate fast red [2]. The fluorescent substrate ELF-97 was utilized to map AP activity to cells on the surface of bone [2]. Figure 3 illustrates a strong expression on the surface of active osteoblastic cells on the endosteal surface of the outer cortical shell of fracture callus as indicated by strong Col3.6GFP reporter activity over a fluorescent mineralization line. In addition, there is weaker activity on the periosteal surface of the repair callus, which we interpret as being a lining cell or inactive osteoblasts based on the absence of Col3.6GFP activity or a mineralization line.

We incubate for 5 minutes and terminate the reaction by rinsing. Subsequently, the section is stained with 4’,6-Diamidino-2-Phenylindole, Dihydrochloride (DAPI). Both steps are done in a batch mode. It is possible to obtain the fast red signal with the Retramethyl Rhodamine Iso-ThioCyanate (TRITC) filter and DAPI with the DAPI filter because the earlier mineralization stains that used these filters are removed during the TRAP staining step.

The section is subsequently stained with Hematoxylin (Polyscience #S216-16oz). This stain does not require dehydration, thus preventing tissue shrinkage. The section is re-imaged using a color camera (AxioCam MRc 5).

### Image analysis of bone

Our image analysis is comprised of six following steps. It is important to note that depending on which instrument and/or methods are used, the specifics of analysis steps may vary. In general, workflows should be easily adjustable and reconfigurable to satisfy many changing requirements.

#### Image assembly

**Figure 4:**
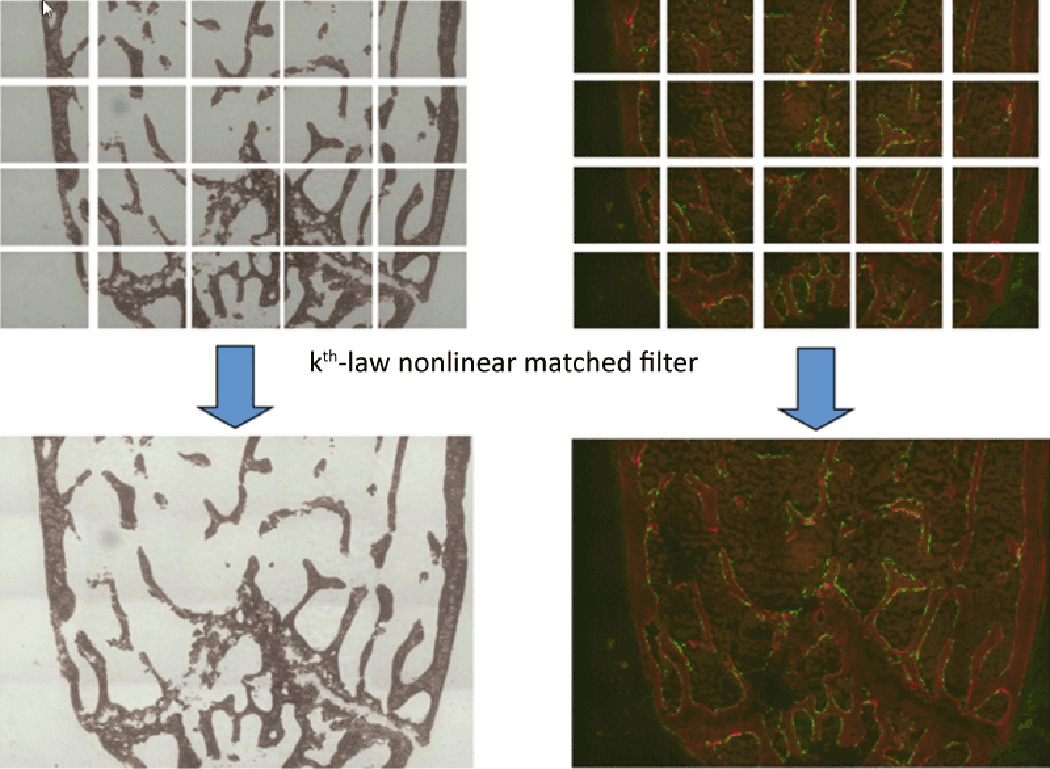
Assembly of Von Kossa images and AC label/GFP images using *k*-th law non-linear matched filtering technique. 15% overlapped adjacent area is searched and matched. 0.3 is chosen for *k* value.

The first optional step is to assemble 2D fractional images directly obtained from the microscope into a whole bone image. Often, the image assembly software embedded in the scanning microscope does not work correctly, so a more efficient algorithm was devised using a *k*-th law nonlinear correlation technique [3]. Adjacent fractional images are scanned to have a 10∼15% overlap. Exact overlap locations can then be found by utilizing the information of the intensity and the outline of the target objects according to the *k* value ranging from 0 to 1. The *k* value can then be used to affect the balancing between the usage of intensity values and the usage of phase information for the object. Our choice was *k*=0.3 for balancing. Figure 4 illustrates image assembly performed on both 20 Von Kossa (VK) stained images and 20 signal images (AC and GFP).

#### Registration of signal images and bone images

**Figure 5:**
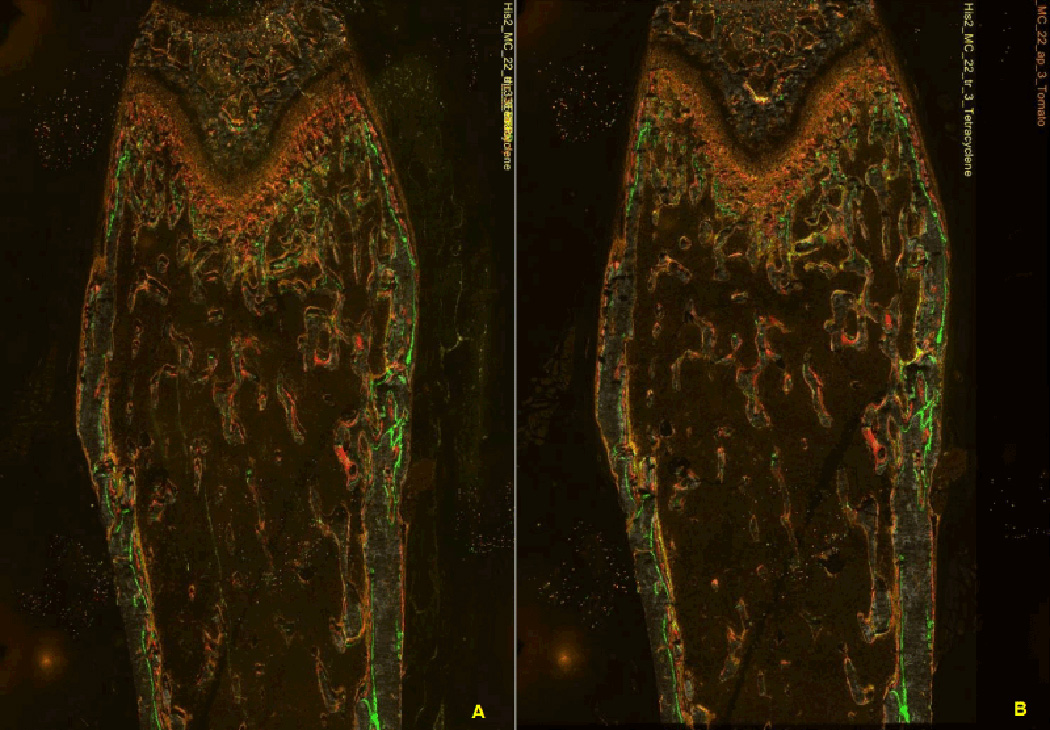
Image registration using red and green fluorescent beads around the femur. Green and red represent mineralized labels of Calcein and alizarin complexone, and yellow represents TRAP positive cells, orange represents AP stained cells. (A) Overlaid image without registration. TRAP and AP positive cells are not aligned. Note that the file annotation on the right upper corner aligned well. (B) Overlaid image after registration. TRAP and AP positive cells are aligned well with bone matrices. The file annotation on the right upper corner tells how far the TRAP image has been off before registration.

The next step is to register the assembled bone image with the assembled osteoblasts (AP stained) and osteoclasts (TRAP stained) image in order to quantify their relationship to the bone surface. For staining TRAP and AP, the sample slide is moved from the scanning stage to the staining stage. Once the slides are TRAP or AP stained on the staining stage and placed back on the scanning stage, due to the moves the slides may not have the same orientation and rotation. Furthermore, AP and TRAP staining processes may have caused shrinkage of the film where the sample was placed on. We need to address this potential shrinkage problem by registering the TRAP and AP stained images to the mineral image. For this registration purpose, we spot 1 µl of green and red fluorescence beads on images next to the bone matrix under the light microscope (see Step 2 in Cryosectioning and bone imaging). We again use *k*-th law nonlinear correlation method to register the images and fuse them together using green and red fluorescence beads in the registration process. Beads found on mineral and label images are used as references, and beads found on TRAP stained image and AP stained image are registered to the references, so that rotational angle, shifts and scale can be accurately estimated. Figure 5 is an example illustrating how effective it is to register TRAP and AP stained images to the mineral/label image using the red and green beads. Before the registration, Figure 5A shows that image annotations on the right upper corner (fixed location) are aligned when TRAP and AP stained image and the mineral/label image are superimposed. Notice that the images do not look aligned. The TRAP signals (represented as yellow) are supposed to be near the surface of the bone, but instead appear inside the bone. After registration, TRAP and AP images are resized, rotated and shifted to be properly aligned to the related mineral image as the correction is illustrated by Figure 5B.

#### Smoothing surface

**Figure 6:**
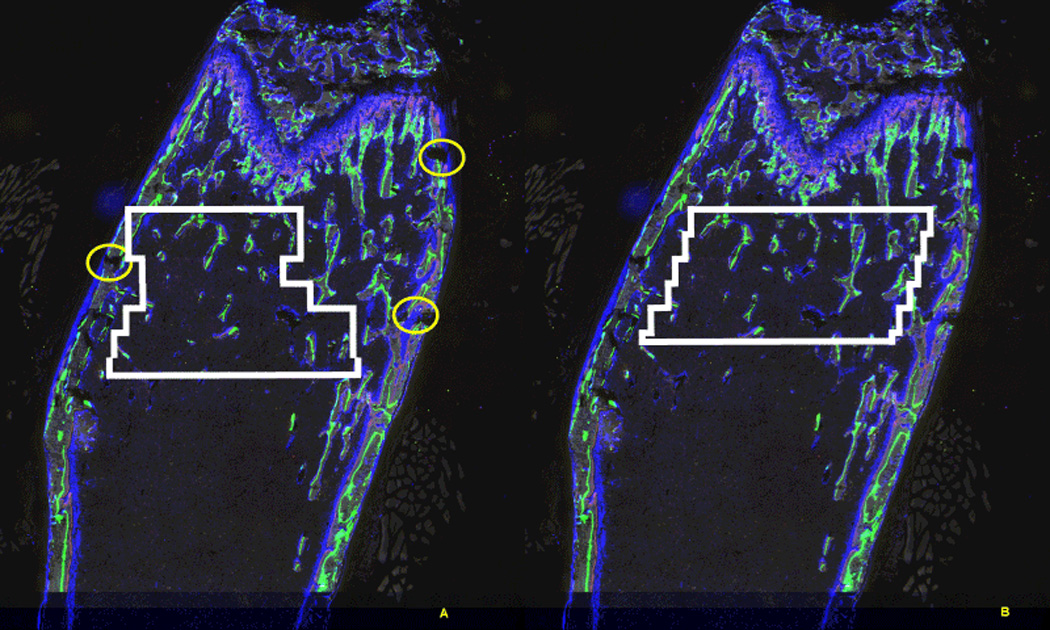
Treatment of the broken cortices. (A) Automatic selection of ROI failed due to the broken cortices marked as circles. (B) After finding the endosteum correctly by deformable matching, ROI is successfully defined.

For various reasons, bone images suffer from ripped or broken surface areas or sites of blood vessel entry. The problem areas with contouring of broken or ripped surfaces should be altered so additional bias in not introduced. By following the circumference of the bone image, a median filter and morphological process (dilation followed by erosion) [4] are used to smooth the surface and to fill the irregularities. Figure 6A shows the case in which automated ROI selection (described in Step 5 below) failed because of three chipped bone cortices. To correct this, we use a deformable matching [5–6] method that robustly finds the endosteal surface. Deformable matching is a method using and elastic template that recursively minimizes the shape distance between the deformed template and calculated contour of the target image. When the damaged bone is recovered with deformable matching, the automated ROI selection becomes less error prone. Figure 6B illustrates the desired selection of ROI in which the ROI boundary is consistently inside the endosteum.

#### Segmentation

**Figure 7:**
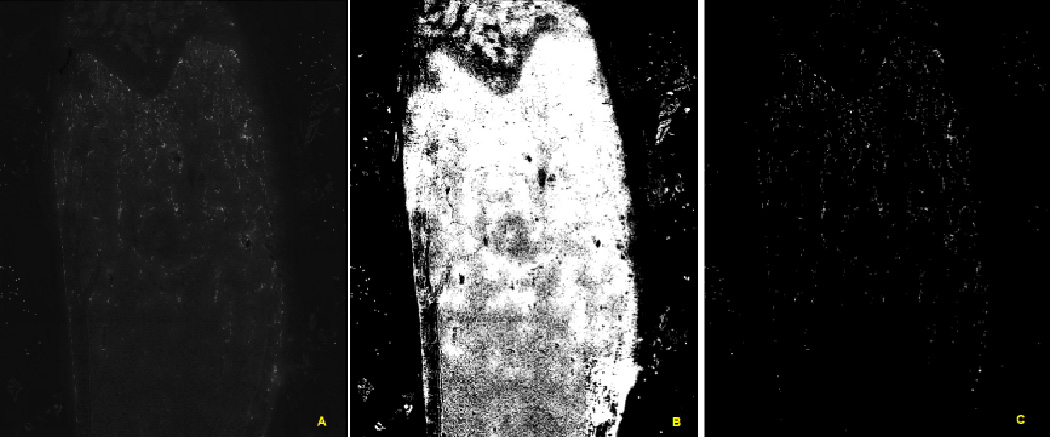
Processing low TRAP signals through cascaded applications of Otsu’s method. (A) Original scanned image (B) Outcome from the application of Otsu’s method on Figure 7A. It did not separate the background pixels inside the femur, and most of the background pixels selected. (C) Outcome from the second application of Otsu’s method on the pixels of Figure 7A selected by Figure 7B. The background pixels are separated and the TRAP signal are selected.

There are four popular approaches to segmentation: threshold techniques [7], edge-based methods [8], region-based techniques [8], and active contour model methods [5]. Our choice has been using threshold techniques because it can be applied to both bone and label/cell images, and it can be implemented without prior threshold criteria or initial values. One of the widely used thresholding methods is Otsu’s method, which relies on a histogram-based technique [9]. A threshold is chosen such that the intraclass variance of the thresholded black and white pixels is minimized while the subsequent interclass variance is maximized. Because only two intensity groups are examined, the separation performance of three or more intensity groups is not optimal. In order to separate the pixel intensities from the backgrounds when there are more than two intensity pixel groups, we applied Otsu’s method iteratively, making the method less affected by the global histogram and mean intensity [10–11]. An example of a double step Otsu’s method is shown in Figure 7. The image in Figure 7A shows a mix of three intensity groups: TRAP signal (highest intensity), background within the marrow space (mid level intensity), and background outside of the femur (lowest intensity). Background intensity levels (within and outside of femur) are different from each other and the background pixel intensity inside of the femur is closer to the TRAP signal intensity when examined globally. The first application of Otsu’s method separates the background pixels outside of the femur, but it does not separate mid level marrow space background pixels from the signal, as shown Figure 7B. The second application of Otsu’s method on the outcome of the first step separates the background pixels within the femur from the TRAP signal as shown in Figure 7C.

#### Select region of interest (ROI)

**Figure 8:**
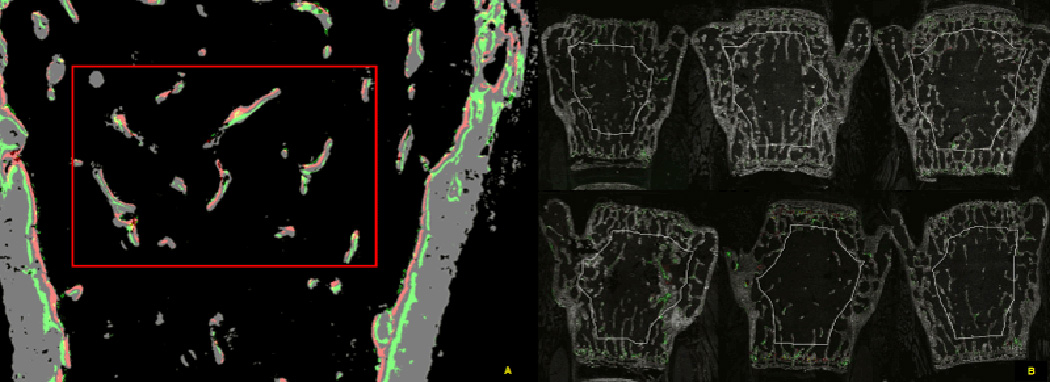
Region of Interest. (A) ROI of a fumur selcted by traditional way. It can be compared with the ROI shown in Figure 6B (rectangular shape vs. tilted shape following the endosteal surface). (B) Example of 6 ROIs selected within 770 µm inside of the endosteal surface of the vertebrae.

In traditional bone histomorphometry, selecting ROI of distal femur and vertebra is determined by the following rules. It can be a rectangular shape or a random shape composed from multiple square fractional images of size 375 µm×375 µm. In distal femur analysis, ROI generally starts 400 µm below the bottom of the growth plate and bounded by the 200 ∼ 300 µm inside of the endosteal surface having a total area of about 2.1 mm^2^ depending on the size of field of view of the microscope. In our automated method, we define the ROI similar to the traditional way by having the same area except the shape of ROI because in a computerized method a much more sophisticated (and hopefully more accurate) shape can be selected. Figure 8A is an example of ROI of a distal femur defined by traditional histomorphometry and Figure 6B illustrates the ROI found by our proposed automated method. Figure 6B shows that we can flexibly produce a finer jagged line of the ROI by following the discovered endosteal surfaces while maintaining the size of the fractional image to 50 µm×50 µm. In vertebra analysis, we set the ROI that is bounded by 770 µm inside of the endosteum of the vertebra, as an example is illustrated in Figure 8B. The distance between the endosteum and the ROI in vertebra and the area chosen should be further studied.

#### Projection and ratios computation

Computing the relationship between bones and labels, or cellular activities associating with bones, can be achieved by projecting labels and cells onto bone surfaces using morphological image processing. Some stained labels and GFP marked or TRAP or AP stained cells are located far from the surface of the bone. Even in a single segment of the label line or cell, some parts are closely located to the bone surface and some are located far from the bone surface. Therefore, it is difficult to find the bone surface corresponding to stained labels or cells. In order to find the precise ratios of the stained labels or cells over the bone surface, they need to be relocated on the surface of the bone surface. By projecting the stained labels or cells onto the trabecular surface, surface ratios of the leading edge (or lagging edge or center line, where tracing center line is tricky in traditional method) of them can be easily analyzed.

**Figure 9:**
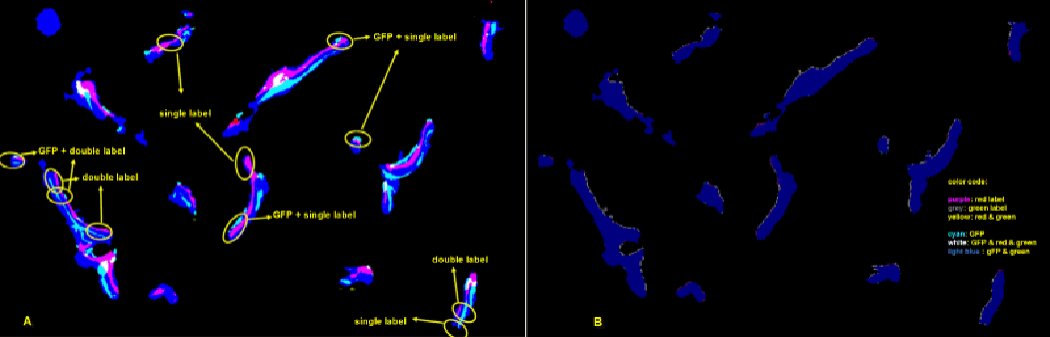
(A) Segmented image inside of ROI from Figure 8A. It shows single label, double label, GFP associated with single label, GFP associated with double label. (B) Projection of the features to the surface of the bone. Each signal is projected onto the surface of the bone by morphological operation, along the calculated surface norm vectors. This projection image is the basis of all the calculated bone surface ratios.

1) Projecting signals onto the trabecular surface is performed by following 4 steps: a) Find the normal vectors of the trabecular surfaces, b) Find the shape and normal vector of a signal: c) Compare the average normal vector of the signal (average direction of the normal vectors of signal) to the normal vectors of the trabecular surfaces, and then determine the direction that matches the both and d) Move the signal along the determined direction one pixel at a time until all of the signal pixels hit the bone surface. Figure 9 illustrates the projection of the signals. Figure 9B shows the projection outcome after the quantification rule is applied to the input image Figure 9A. It should also be noted that the projection not only distinguishes single label, double label, GFP, and GFP associated with each mineralizing label but also makes the signals ready for calculating various ratios.

2) Inter-label thickness - If there are two labels, inter-label thickness is measured as follows. Distance between the surface of the trabecula and the first mineralization line (first label distance) is measured by projecting the first mineralization line onto the trabecular surface, measuring the pixel distances between the mineralization line and the surface of the trabecula. The distance between the trabecular surface and the second mineralization line (second label distance) can also be measured in the same way. The inter-label thickness is the difference between the first label distance and the second label distance. This can be done using leading edge distance (distance between the leading edge and the trabecular surface), or mid-point distance (average of leading edge distance and the lagging edge distance (distance between the lagging edge and the trabecular surface)). Leading edge distance and mid-point distance are measured in pixel distance while signal projection is performed. Leading edge distance is number of movement in projection step 4, where the signal first hits the bone surface, and the mid-point distance is the distance where the mid-points of the signals meet the bone surface. The pixel distances are converted into Euclidean distances.

3) Cellular activities-In general, we follow the measurement notations defined in [12–14] for static and dynamic histomorphometry. However, our method produces extra measurements that are not defined in the traditional manual bone histomorphometry, such as individual single label surface ratios L2/BS and L1/BS, where L2/BS and L1/BS represent second injected (2 days before sacrifice) and first injected (7 days before sacrifice) label surface ratio, respectively. In addition, new measurements related to cellular activities are introduced for the first time in this paper.

a) Osteogenic activity - For osteogenic activity, we introduce AP/BS to measure all AP surfaces (AP/BS), AP associated with the second mineralization line (AP_L2/BS=active osteoblast) and AP not associated with either mineralization line (AP_only/BS=bone lining or inactive osteoblast).

b) Osteoclastic activity - For osteoclastic activity, we introduce TRAP/TV in which we separate the total TRAP activity within the ROI, and the proportion of the total TRAP that is on the bone surface (TRAP_on/TRAP) as opposed to being within the marrow space. AP and TRAP positive cells within 25.6 µm (67.4 µm for rats) of the bone surface (AP/BS and TRAP/BS) are measured. Probably more relevant to traditional histomorphometry is the distribution of TRAP on the bone surface that is not associated with a mineralization line (TRAP_ only/BS=eroding surface) or with ongoing mineralization (TRAP_L2/ BS). Table 2 lists the measured and calculated parameters used in the analysis.

The image analysis for automatic histomorphometry is implemented in Matlab (R2010a, 64-bit). Although the operational time per sample majorly depends on the trabecular mass and the amount of the mineralization line within ROI, it takes about 20 minutes to compute all the ratios of one femur when the analysis is done on a single medium-power PC (dual core 3GHz zeon processor with 4GB memory, 64-bit operating system).

## Results

### Imaging and image analysis workflow

**Figure 10:**
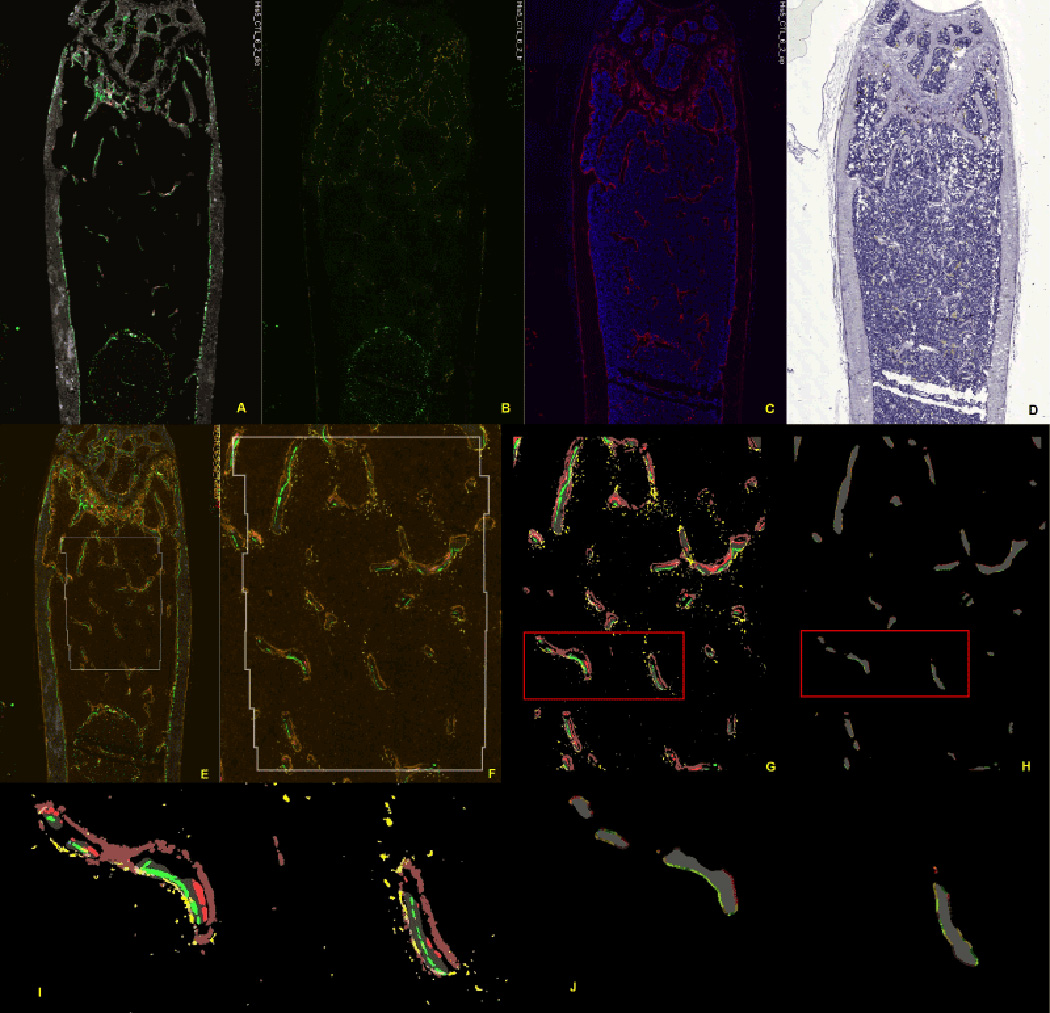
Work flow of image capture and the subsequent detection of the fluorescent signals. (A–D) represent microscope steps, while (E–H) are image processing steps. (A) Scan 1 captures mineralized bone and the fluorescent mineralization lines. (B) Scan 2 records the distribution of TRAP activity but loses the mineralization lines. (C) Scan 3 is performed after AP and DAPI staining. (D) Traditional hematoxylin stain for morphologic considerations, but this section is not part of the analysis. (E) Alignment of all the fluorescent images files into a single image stack and selection of the region of interest in the context of the whole bone. This step corrects for minor variation is rotation and shrinkage of the tape. (F) Enlarged view of the ROI prior to computer rendering. (G) Computer representation of the fluorescent features that are captured by the thresholding algorithms. (H) Projection of the features to the surface of the bone. This image is the basis of all the calculated measurements that are related to the bone surface. (I) and (J) are magnification of the boxed area of G and H. The enlarged figure demonstrates two trabeculae (grey signal) with bone formation (maroon colored AP overlying a red and green label) on the right side and resorption (yellow TRAP over non-mineralizing surface) on the opposite side.

The cryosections are imaged in four separate settings, each of which generates a tiled image set with one specific signal designed for each analysis feature. Figure 10A–D illustrates image set 1 (calcein blue=mineralized bone; calcein green=first mineralization label; alizarin complexone=second mineralization label; other labels for GFP-emitting cells can be captured at this stage or later stages), set 2 (Yellow=ELF-97; note that most of the signals from set 1 are lost in set 2), set 3 (red=fast red for AP activity, DAPI for bone marrow and osteocytes), and set 4 (hematoxylin=cellular elements for visual inspection but not used in the analysis). A minimum of 6 fluorescent files (calcein blue, calcein green, AC, ELF-97, fast red and DAPI) are submitted for signal thresholding and analysis per section. The analysis is based on 8 animals in a group, and from which three sections per bone are sampled. The image analysis program generates an additional 3+ files documenting the computational steps (Figure 10E–J). They include the merged and pseudo-colored image showing the computer selected ROI (Figure 10E–F), the thresholded signals within the ROI (Figure 10G, H), and the projected signals to the bone surface (Figure 10H, J).

### Technical validation of the image analysis

**Figure 11:**
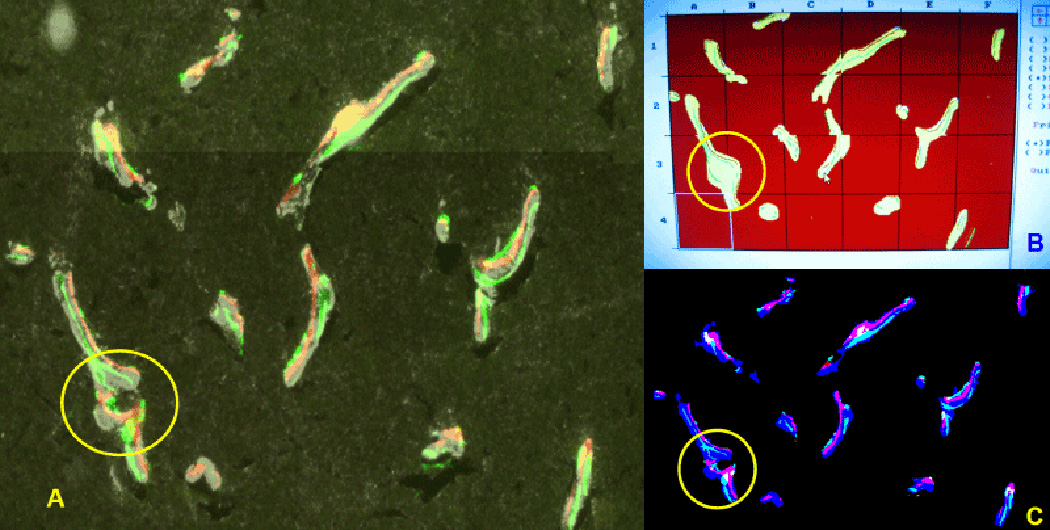
Comparison between manual and automated histomorphometry shown in Figure 8A. (A) Microscope view of DIC image with two labels (red and green) and GFP within ROI shown in Figure 8A. (B) A snap shot of signals of traditional manual analysis tracing. It shows overly smooth bone surfaces. Circled area in (A) is filled up. (C) Automated segmentation of DIC image with labels and GFP in automatic process as shown in Figure 8A. It maintains the original shape of circled area shown in (A).

For the purpose of validation, we compared static and dynamic measurements of the fluorescent signals within the same cryosection obtained from our automated image analysis program with the ones obtained by the manual method using a commercial analysis platform (OsteoMeasure). We have relied on Dr. Boguslawa Koczon-Jaremko who was trained by the renowned bone histomorphometry expert Dr. Gloria Gronowicz of University of Connecticut Health Center. Starting with the same image (Figure 11), Dr. Koczon-Jaremko independently carried out the analysis using the conventional method using OsteoMeasure. Figure 11 illustrated the images used for the comparison. Figure 11A is the microscopic view of the ROI, Figure 11B is the actual screen capture of the commercial analysis platform, and Figure 11C is the segmented image of the same ROI using the proposed method. The circled area in Figure 11B illustrates the general practice that the human operator fills the crevice to make the bone surface to be smooth. In contrast, we show in Figure 11C that the automated method does not fill the gap. The reason for the filling in the former approach is for the measuring convenience, but it clearly suggests potential inaccuracy risks, particularly, if such filling is done in multiple locations. Table 3 shows that detection of GFP positive bone surface cells in which single and double labeled surfaces and inter-label thickness are highly correlated as is the detection of regions of bone mineral. Thus when the fluorescent signals are strong and distinctive, the two methods correlated very well. It takes about 40 minutes for an experienced technician to analyze the bone using the commercial analysis platform, while it takes about half of that time for a computer to analyze the same section. At a glance the difference may not seem big, but two issues should be taken into account in the comparison: (i) when the size of the samples increases (e.g., hundreds of images) the tedium and the cost of the manual analysis could become a serious issue, and (ii) the speed of automated processing in this comparison is somewhat artificial because use of a powerful cluster computer would reduce the processing drastically (e.g., 2 minutes as opposed to 20 minutes).

### Biological validation

**Figure 12:**
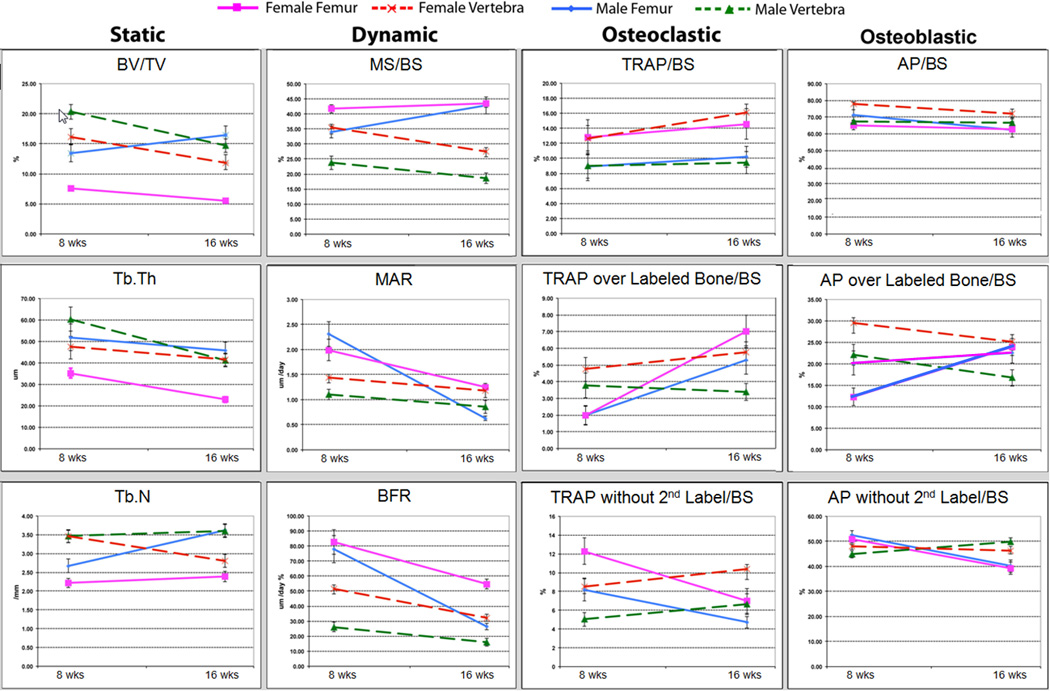
Summary data for vertebra and femur from male and female C57Bl/6J mice at 8 and 16 weeks of age.

To demonstrate the performance of our cryohistology and image analysis approach in a common experimental situation, we utilized a well-studied mouse line where numerous publications that containing static, dynamic and cellular histomorphology data exist for comparison. We purchased 16 male and 16 female C57Bl/6J mice at 4 weeks of age from The Jackson Laboratory. We chose to make a direct sex and age specific comparison at two commonly sampled sites (distal femur and vertebra) because distinct dimorphic differences have been observed, but no study that we could find has look at these common variables with the same experimental groups. Groups of 8 from each gender were maintained to 8 and 16 weeks of age and injected with calcein green and AC at 7 and 2 days prior to sacrifice. The femur and lumbar vertebrae 3–5 were harvested for processing and analysis. The analysis results of femur and vertebrae of male and female C57Bl/6J mice for 8 and 16 weeks of age are shown in table 4, and graphical illustrations of the major measurements are depicted in Figure 12. We have used 3 sections from each femur and vertebra which are as close as to the center in the longitudinal direction. The values of three sections per bone section are averaged and mean value from each section is used to calculate statistical significance between two contrasted measurements by the paired t test [15].

#### Static histomorphometry

As expected, female C57Bl/6J mice have diminished trabecular mass (BV/TV) relative to the male in distal femur (7.5 female vs. 14.0 male at 8 weeks and 5.5 female vs. 16.5 male at 16 weeks) and in the vertebra (16.2 female vs. 20.5 male at 8 weeks and 11.8 female vs. 14.4 male at 16 weeks) due primarily to a reduction in the trabecular thickness at both time points [16]. Thus females loose bone mass between 8 and 16 weeks of age in both bone sites, which is a trend that will continue over the next 4–6 months while males remain stable in the femur but loose in the vertebra [16]. The higher trabecular content in vertebra versus distal femur has been noted previously [17–8].

#### Dynamic histomorphometry

The larger loss of trabecular bone in the females occurs in the face of higher BFR in both locations and at both ages (Femur: 0.82 female vs. 0.78 male at 8 weeks and 0.54 female vs. 0.27 male at 16 weeks; Vertebra: 0.51 female vs. 0.29 male at 8 weeks and 0.32 female vs. 0.16 male at 16 weeks). The difference in BFR is primarily related to a greater mineralizing surface at 8 weeks in females (Femur 41.8 female vs. 33.9 male; Vertebra 35.6 female vs. 25.1 male) while the MAR contributes a more prominent effect at 16 weeks in the femur (1.25 female vs. 0.63 male). Thus at both ages, the experiment outcome validates the general understanding that female mice have a significantly higher bone turnover in both femur and vertebra than male mice primarily due to more active osteogenic surfaces. However the exception that is observed in the MAR as opposed to MS/BS seen in females at 16 weeks was unanticipated but was consistent with measurements of active osteogenic surfaces as described below.

#### Osteoclast activity

Overall, the osteoclast analysis in the vertebra and femur were internally consistent. TRAP/BS was higher in the females in both sites and the level did not change with age (14.5 female vs. 10.4 male at 8 weeks, 13.8 female vs. 9.8 male at 16 in the femur; 13.6 female vs. 8.7 male at 8 weeks, 16.2 female vs. 10.3 male at 16 weeks in the vertebra). Differences in the fraction of TRAP associated either with or without a mineralizing surface were similar in the vertebra but shifted toward a higher proportion of the activity in the mineralizing fraction of the bone surface. Given the high variance of the TRAP measurements, particularly those associated with a mineralizing surface, these changes may be within experimental error. What is clear is that by 16 weeks of age, all measures of TRAP activity are significantly elevated in females in both bone sites.

#### Osteoblast activity

The AP measurement showed consistent changes in both sexes in the vertebra but somewhat contradictory changes in the femur. There was a general downward but non-significant trend between 8 and 16 weeks in total AP/BS in the vertebra and femur in both sexes (vertebra: 78.0 female vs. 66.9 male at 8 weeks and 71.4 female vs. 67.1 male at 16 week; femur: 71.2 female vs. 64.8 male at 8 weeks and 63.8 female vs 63.1 in the males at 16 weeks). Using the measurement of AP over a mineralizing surface (active osteoblast), there was significantly higher osteogenic activity in female animals in vertebra at 8 and 16 weeks and in femur at 8 weeks (vertebra: 30.0 female vs. 22.0 male at 8 weeks and 24.8 female vs. 17.1 male at 16 week; femur: 20.2 female vs. 12.3 male at 8 weeks). However by 16 weeks there was similar level of osteogenic cells in the two sexes (24.5 vs 23.0), and this observation is consistent with the similar MS/BS measurement made by dynamic histomorphometry. No dimorphic or age related change was evident in the inactive osteoblast population (AP over non-mineralizing surface). There are many possible explanations for the unanticipated 16 week old femur measurements that relate to skeletal maturation achieved in the male between 8–16 weeks of age, and genetic determinants of trabecular bone between vertebral and distal femur.

## Discussion

### Validation

Our project was initiated to determine if the histological advantages for cyrohistology of non-decalcified bone that were initially developed for studying GFP reporter activity in mineralized tissues could be adapted for bone histomorphometry in non-transgenic animals. The key question for the value of this new approach to bone histomorphometry is whether the generated data is sufficiently reliable and biologically relevant to be accepted by the skeletal biology community. Unfortunately a direct comparison of the two approaches has not been possible. In the sample we have examined, the background fluorescence of methyl methylate embedded sections is higher and the mineralization lines are less intense than the cryosections and cannot be resolved by our image analysis procedure. Because our institution no longer supports a bone histomorphometry core, we do not have access to affordable traditional analysis. Core services at other academic institutions have not had the flexibility to provide us with paired samples, and commercial analysis sites are prohibitively expensive.

A. Direct comparison-To overcome these logistical problems, the validation question was examined in two ways. First, we compared and confirmed that the measured data from our automated method and the one from the traditional method of the same samples were remarkably similar. It indicates that the signal discrimination and measurement algorithms that we developed are emulating the assessment practices of a skilled technician.

B. Literature comparison-Second, we examined whether the biological conclusions made in the literature using traditional histomorphometry can be replicated by our automated methods. It appears that the static measurements parallel those made by µCT in terms of the trabecular bone volume differences between vertebral and femur, age and most importantly sex. The rapid loss of trabecular bone in the distal femur of female mice makes vertebra a more desirable choice for assessing dynamic and cellular activities along trabecular bone. The variance of the histological approach is inferior to µCT (see table 1 in supplementary data) but the sample size of 8 or greater makes a statistical study meaningful. Our dynamic measurements of bone formation also are consistent with published observations. Higher bone turnover in female animals relative to males has been found and is generally assumed to be a direct effect to the effect of sex hormones on osteoclastic activity, which secondarily results in a higher level of bone surfaces with active mineral deposition. The variance in measuring the mineralization lines from which the BFR is calculated is particularly small making the dynamic measurement the most statistically powerful assessments of our analysis. One finding from the L2/BS and L1/BS data that may be of significance is the difference between the two measurements is always greater in the female animals. This difference may reflect a greater loss of the first label due to osteoclastic activity and greater compensatory osteogenic response as seen in the second label. This type of calculation may prove useful for assessing *in vivo* osteoclastic activity between a test and control experimental situation. This approach has been employed using *in vivo* imaging of fluorescently tagged bisphosphonate probes [19].

### Cellular analysis

The cellular analysis is based of enzymatic surrogate of osteoclast and osteoblasts. In the case of osteoclasts, the fluorescent TRAP substrate identified both multi and unicellular cells within the osteoclast lineage both adjacent to the bone surface and within the bone marrow. In our current study, we find the measurement of TRAP activity in proximity of the bone surface, particularly those not associated with a mineralizing surface, most relevant to the osteoclast number found in published studies [20–22]. While it is possible to limit the analysis to signals of particular size or signals that also contain multiple DAPI positive nuclei, we felt that a more global view of the total distribution of TRAP positive cells could be more informative to investigators studying osteoclast biology.

The use of AP activity for distinguishing cells of the osteogenic lineage from other bone surface associated cells has proven to be a useful histological feature. Currently, the visual identification of osteoblasts as bone surface cell with a cuboidal morphometry is well accepted. However, visual methods for excluding other bone surface cells such as osteomacs [23], mononuclear osteoclasts [24] or a bone lining cells are not well established. Our experiments suggest it is possible to discriminate some of these ambiguities of cells on the bone surface. We find that the measurement of AP activity in association with the bone surface undergoing active mineralization activity is results in a value that is higher that reported on morphological criteria [20–22], probably because the later does not identify metabolically active osteogenic cells. Because the AP/mineralizing surface is a functional biological measurement, similar to the way osteoclasts are identified, it is also less subject to observer judgment. Furthermore we postulate that AP positive cells that are not undergoing active mineralizing activity can be identified as bone lining cells capable of regaining osteogenic activity. This definition will need further verification, but based on our observations in early fracture healing, AP positive and mineralization negative periosteal cells with these histological properties will rapidly develop GFP reporter activity of an osteogenic cell and begin to deposit a mineralizing matrix. When these AP accessing rules are applied to our histomorphometic analysis, we observe that the proportion of bone surfaces with an AP positive signal remain relatively constant with age, but is minimally higher in female animals. When the activity is partitioned between active osteoblast vs bone lining osteoblasts, female animals have a higher proportion of metabolically active osteoblast while the metabolically inactive cell of the osteogenic lineage is similar in both sexes. However there is one exception that was found in the distal femur at 16 weeks. Although the bone formation rate is still higher in the female animals, this is accomplished not by recruiting more active osteoblasts to the bone surface but by increasing the matrix formation rate. This conclusion is consistent with independently derived dynamic and AP-based cellular data. Clearly more reliable measurements of mineralizing and cellular activity will be achieved once a stable equilibrium of bone turnover has been reached by 3–4 months of age. Similar results of male versus female bone formation and turnover can be amply found in the literature [25–30] although a change during ageing and sexual maturation has not been specifically studied.

### Power analysis

Table 5 is excerpted from the power analysis [31] to demonstrate the fold difference that the method can detect when there are 6 mice per groups with a confidence limit of 90%. It demonstrates that the dynamic measurements are the most sensitive to change at all ages, while the cellular measurements of the vertebra at 16 weeks are the most stable and sensitive for differences in the dynamic behavior of bone between males and females. Full power analysis data is shown in supplementary table 2– supplementary table 5.

## Conclusion

We have demonstrated that technological advances in biology, materials science and image processing can enable an automated way of performing bone histomorphometry. In the traditional way, histomorphometric measurement is performed by computer-aided manual tracing of the staining signals. Our approach combines cryosectioning and fluorescence-based histology followed by automated image processing capable of generating quantification data from the signals. Manual tracing can be replaced with the proposed automated image processing. Our automated method offers multiple advantages over the traditional method. The automated method is by far more expeditious and cost effective. It allows obtaining additional measurements (e.g., individual single label surface ratios) which are not typically assessed in the traditional approach. Ultimately, we envision that the fluorescence-based histology can be expanded to detect osteoid surfaces [32] and osteocyte density [33] as well as detection of other cellular activities revealed by fluorescent substrates used for *in vivo* imaging studies [19]. More importantly, our method minimizes—if not eliminating completely—the observer bias in the analysis. The image processing routines apply the same signal interpretation rules, thus offering opportunities for meaningful comparison analysis of outcomes obtained from independent laboratories. This automated method could serve to form a larger foundation in which high-throughput bone histomorphometry is used by laboratories with specific expertise, to collaborate on new and challenging large-scale studies involving gene knockout, tissue engineering and biomaterial development.

## Figures and Tables

**Table 1:** Effects of exposure time to the measurements.

parameters	high exposure	auto exposure	reduced exposure
L2/BS	38.73%	20.38%	33.06%
sLS/LS	53.87%	29.66%	42.37%
LS/BS	61.50%	36.95%	50.28%

**Table 2:** Definition of Histomorphometric Measurements.

Static Measurements		
BV/TV	M	Bone volume per total volume
Tb.Th	M	Trabecular thickness
Tb.N	C	Trabecular number
Tb.Sp	C	Trabecular separation
Dynamic Measurements		
L2_only/BS	M	Second injected label per bone surface
L1_only/BS	M	First injected label per bone surface
sLS/BS	M	Single label surface per bone surface
dLS/BS	M	Double label surface per bone surface
LS/BS	C	Labeled surface (single or double) per bone surface
MS/BS	C	Mineralizing surface per bone surface
sLD/LS	C	Proportion of labeling surface that are single label
dLS/LS	C	Proportion of labeling surface that are double label
dLS/sLS	C	Ratio of double to single labeled surface
Ir.L.Th	M	Interlabel thickness
MAR	C	Mineral apposition rate (µm/day)
BFR	C	Bone formation rate (MAR × (MS/BS))
Cellular Measurements		
AP/BS	M	Fraction of bone surface with AP label
AP_R/BS	M	Fraction of bone surface with AP activity and the second mineralization label
AP_only/BS	M	Fraction of bone surface with AP activity but without the second mineralization label
TRAP/BS	M	Proportion of the bone surface with proximal TRAP activity (25.6 µm distance)
TRAP_R/BS	M	Proportion of the bone surface with proximal TRAP activity and the second mineralization label
TRAP_only/BS	M	Proportion of the bone surface with proximal TRAP activity but without the second mineralization label
TRAP/TV	M	Total TRAP activity within the selected ROI
TRAP on/TRAP	C	Proportion of total TRAP activity that is adjacent to the bone surface

M=measured value, C=calculate value from other measurements

**Table 3:** Comparison of the feature measurement methods on the same histological section.

Analytic method	GFP/BS(%)	sLS/BS(%)	dLS/BS(%)	MS/BS(%)	Ir.L. Th(µm)	MAR(µm/day)	BV/TV(%)
Traditional	7.91	14.95	22.40	37.35	15.38	2.20	8.61
Automated	8.32	12.32	26.13	32.29	15.36	2.19	8.60

**Table 4:** Histomorphometric measurements of male and female C57Bl/6J mice taken at 8 and 16 weeks of age (units are % except MAR and BFR)

Dynamic - Femur														
**8 wks**		L2/BS	L1/BS	L2_only/BS	L1_only/BS	sLS/BS	dLS/BS	LS/BS	MS/BS	sLS/LS	dLS/LS	dLS/sLS	MAR(µm/ day)	BFR(µm^3^/ µm^2^/day)
**Male**	**mean**	43.97	23.92	30.64	10.59	41.24	13.32	54.56	33.94	75.99	24.01	32.43	2.31	0.78
	**std**	6.54	5.83	3.98	3.69	4.09	3.89	7.00	5.28	4.40	4.40	7.93	0.53	0.210
**Female**	**mean**	55.12	28.38	38.02	11.28	49.29	17.10	66.39	41.75	74.38	25.62	35.57	1.99	0.82
	**std**	6.51	5.31	5.88	3.47	3.14	4.37	3.83	3.80	5.39	5.39	10.51	0.41	0.172
**16 wks**	**p**	9.1E-03	1.7E-01	1.6E-02	7.3E-01	3.0E-03	1.1E-01	6.8E-03	1.4E-02	5.5E-01	5.5E-01	5.4E-01	2.6E-01	6.8E-01
**Male**	**mean**	51.77	35.95	29.90	14.08	43.98	21.87	65.85	43.86	67.05	32.95	49.83	0.62	0.27
	**std**	6.14	4.78	3.85	2.35	3.24	3.94	5.94	4.77	3.67	3.67	8.28	0.07	0.03
**Female**	**mean**	53.75	33.38	33.97	13.60	47.57	19.78	67.35	43.57	70.75	29.25	42.14	1.25	0.54
	**std**	4.00	2.56	2.16	2.04	2.91	2.84	3.76	3.00	3.35	3.35	6.60	0.14	0.08
	**p**	4.6E-01	2.1E-01	2.4E-02	6.7E-01	3.5E-02	2.4E-01	5.6E-01	8.8E-01	5.4E-02	5.4E-02	6.0E-02	5.2E-07	6.1E-06
**Static - Femur**						**Osteogenic - Femur**				**Osteoclastic - Femur**				
**8 wks**		BV/TV	Tb.Th	Tb.N	Tb.Sp		AP/BS	AP_L2/ BS	AP_only/ BS		TRAP/ BS	TRAP_ L2/BS	TRAP_ only/BS	TRAP_on/ TRAP
**Male**	**mean**	14.04	54.12	2.70	330.60	**mean**	64.80	12.28	52.88	**mean**	10.37	2.19	8.17	89.69
	**std**	3.10	12.90	0.31	37.39	**std**	4.89	4.71	6.98	**std**	3.62	1.04	3.33	3.88
**Female**	**mean**	7.47	34.15	2.23	432.91	**mean**	71.22	20.22	51.00	**mean**	14.51	2.22	12.29	82.60
	**std**	1.13	2.62	0.31	68.74	**std**	8.51	7.45	4.72	**std**	5.08	1.39	3.95	6.36
**16 wks**	**p**	2.6E-03	1.2E-02	1.8E-02	4.3E-03	**p**	9.1E-2	2.6E-2	5.3E-01	**p**	8.3E-2	9.6E-01	4.3E-02	2.0E-02
**Male**	**mean**	16.48	45.83	3.62	235.55	**mean**	63.18	23.00	39.74	**mean**	9.77	5.03	4.92	88.52
	**std**	3.81	7.55	0.38	35.16	**std**	11.87	6.89	7.68	**std**	3.55	1.91	2.02	2.71
**Female**	**mean**	5.51	22.92	2.39	408.57	**mean**	63.78	24.51	38.81	**mean**	13.86	6.88	7.79	74.61
	**std**	0.93	1.89	0.31	59.37	**std**	8.68	2.32	8.65	**std**	5.48	2.44	3.67	7.56
	**p**	5.3E-05	3.6E-05	7.6E-06	1.7E-05	**p**	9.4E-01	7.0E-01	5.6E-01	**p**	2.6E-01	2.8E-01	3.0E-01	2.8E-02
**Dynamic - Vertebra**														
**8 wks**		L2/BS	L1/BS	L2_only/BS	L1_only/BS	sLS/BS	dLS/BS	LS/BS	MS/BS	sLS/LS	dLS/LS	dLS/sLS	MAR(µm/ day)	BFR
**Male**	**mean**	36.43	13.85	30.63	8.05	38.67	5.80	44.47	25.14	87.44	12.56	14.59	1.19	0.29
	**std**	6.34	4.61	4.77	2.72	4.78	2.41	6.95	4.62	3.35	3.35	4.49	0.23	0.04
**Female**	**mean**	47.32	23.78	34.15	10.61	44.76	13.17	57.93	35.55	77.39	22.61	29.52	1.44	0.51
	**std**	3.49	2.53	3.29	1.58	2.47	2.07	2.67	2.04	3.18	3.18	5.44	0.14	0.06
**16 wks**	**p**	2.9E-03	7.0E-04	1.3E-01	5.5E-02	1.5E-02	3.9E-05	1.6E-03	5.6E-04	5.7E-05	5.7E-05	6.0E-05	3.0E-02	3.2E-06
**Male**	**mean**	24.05	13.27	21.01	10.23	31.24	3.05	34.28	18.66	91.98	8.02	8.93	0.92	0.16
	**std**	5.98	3.91	4.49	2.35	6.30	1.85	7.86	4.76	3.77	3.77	4.59	0.18	0.05
**Female**	**mean**	35.42	19.43	28.21	12.22	40.43	7.21	47.64	27.42	85.07	14.93	17.66	1.18	0.32
	**std**	6.11	2.56	4.99	2.57	3.51	1.33	4.78	3.04	1.34	1.34	1.86	0.10	0.04
	**p**	2.1E-03	2.9E-03	9.0E-03	1.3E-01	4.2E-03	1.9E-04	1.6E-03	9.0E-04	9.4E-04	9.4E-04	6.9E-04	5.7E-03	1.3E-05
**Static - Vertebra**						**Osteogenic - Vertebra**				**Osteoclastic - Vertebra**				
**8 wks**		BV/TV	Tb.Th	Tb.N	Tb.Sp		AP/BS	AP_L2/ BS	AP_only/ BS		TRAP/ BS	TRAP_ L2/BS	TRAP_ only/BS	TRAP_on/ TRAP
**Male**	**mean**	20.51	61.28	3.45	330.60	**mean**	66.90	22.06	45.18	**mean**	8.68	3.64	4.90	87.97
	**std**	2.50	8.24	0.24	37.39	**std**	9.41	6.96	5.46	**std**	3.27	1.40	2.61	3.83
**Female**	**mean**	16.15	47.56	3.46	432.91	**mean**	78.15	30.05	48.41	**mean**	13.65	5.13	8.68	82.86
	**std**	3.27	10.81	0.29	68.74	**std**	2.10	2.37	2.56	**std**	3.86	1.60	2.57	82.86
**16 wks**	**p**	1.0E-02	1.4E-02	9.3E-01	4.3E-03	**p**	1.9E-02	2.2E-02	8.3E-02	**p**	1.5E-02	6.7E-02	2.7E-02	8.0E-02
**Male**	**mean**	14.44	40.72	3.57	235.55	**mean**	67.10	17.11	49.81	**mean**	10.30	3.66	6.52	84.58
	**std**	3.08	7.73	0.31	35.16	**std**	7.25	4.78	5.49	**std**	2.41	0.80	2.85	4.19
**Female**	**mean**	11.83	41.65	2.80	408.57	**mean**	71.14	24.84	46.94	**mean**	16.22	5.84	10.34	81.77
	**std**	3.23	5.23	0.48	59.37	**std**	7.66	4.59	4.68	**std**	2.44	1.45	1.73	5.70
	**p**	1.2E-01	7.8E-01	2.7E-03	1.7E-05	**p**	3.0E-01	5.3E-03	8.0E-01	**p**	2.5E-04	3.4E-03	2.1E-05	2.8E-01

**Table 5:** Sensitivity of the fluoresence-based histomorphometry calculated from the power analysis. Fold difference between a test and control group that can be detected based on 6, 8 10 or 12 animals per group and a confidence level of 90%.

6 mice	MS/BS	MAR	BFR	BV/TV	AP/BS	AP_L1/BS	AP_only/BS	TRAP/BS	TRAP_only/BS
8wk Femur	1.4	1.6	1.7	1.6	1.3	2.4	1.3	2.4	2.5
16wk Femur	1.3	1.3	1.4	1.6	1.5	1.7	1.5	2.5	2.6
8wk Vertebra	1.4	1.5	1.4	1.5	1.4	2.1	1.2	2.3	2.1
16wk Vertebra	1.7	1.5	1.8	1.7	1.3	1.4	1.3	1.6	1.7
8 mice	MS/BS	MAR	BFR	BV/TV	AP/BS	AP_L1/BS	AP_only/BS	TRAP/BS	TRAP_only/BS
8wk Femur	1.3	1.5	1.6	1.5	1.3	2.1	1.2	2.1	2.2
16wk Femur	1.2	1.3	1.3	1.5	1.4	1.6	1.4	2.2	2.2
8wk Vertebra	1.3	1.3	1.4	1.4	1.3	1.8	1.2	2.0	1.9
16wk Vertebra	1.6	1.4	1.6	1.6	1.3	1.2	1.2	1.5	1.5
10 mice	MS/BS	MAR	BFR	BV/TV	AP/BS	AP_L1/BS	AP_only/BS	TRAP/BS	TRAP_only/BS
8wk Femur	1.3	1.4	1.5	1.4	1.2	1.9	1.2	1.9	2.0
16wk Femur	1.2	1.2	1.3	1.4	1.4	1.5	1.4	2.0	2.0
8wk Vertebra	1.3	1.3	1.3	1.4	1.3	1.7	1.2	1.8	1.8
16wk Vertebra	1.5	1.3	1.5	1.5	1.2	1.2	1.2	1.4	1.5
12 mice	MS/BS	MAR	BFR	BV/TV	AP/BS	AP_L1/BS	AP_only/BS	TRAP/BS	TRAP_only/BS
8wk Femur	1.2	1.4	1.4	1.4	1.2	1.8	1.2	1.8	1.9
16wk Femur	1.2	1.2	1.2	1.4	1.3	1.4	1.3	1.9	1.9
8wk Vertebra	1.3	1.3	1.3	1.3	1.2	1.6	1.2	1.7	1.7
16wk Vertebra	1.4	1.3	1.5	1.4	1.2	1.2	1.2	1.4	1.4

## Abbreviations

AC: Alizarin Complexone
AP: Alkaline Phosphatase
BFR: Bone Formation Rate
DAPI: 4’,6-Diamidino-2-Phenylindole, Dihydrochloride
ELF-97: Enzyme-Labeled Fluorescence-97
GFP: Green Fluorescent Protein
MAR: Mineral Apposition Rate
ROI: Region Of Interest
TRAP: Tartrate Resistant Acid Phosphatase
TRITC: Retramethyl Rhodamine Iso-ThioCyanate
VK: Von Kossa
